# Agentic AI for Clinical Outcomes Research, Population Health Management Analyses With Large Administrative Databases, and Generating Epidemiological Estimates of Diseases: Feasibility and Validation Study

**DOI:** 10.2196/100284

**Published:** 2026-09-30

**Authors:** Daniel Frisch, Michael Fronstin, Carla Zema

**Affiliations:** 1 Bruce & Robbi Toll Heart and Vascular Institute Division of Cardiology Thomas Jefferson University Philadelphia, PA United States; 2 Vireo Strategies Philadelphia, PA United States; 3 Zema Consulting Athens, AL United States

**Keywords:** agentic artificial intelligence, agentic AI, clinical outcome research, population health, administrative data, prevalence, real-world evidence, artificial intelligence, AI

## Abstract

**Background:**

There is tremendous enthusiasm for the use of AI in health care because of the ability to analyze existing data for preventative, diagnostic, and treatment support. Agentic AI can feasibly provide access to large real-world datasets for the generation of real-world evidence for health care and clinical applications to health care providers, researchers, and administrators without access to large analytic programming resources.

**Objective:**

The objective of this study was to understand the feasibility of using agentic AI for clinical outcome research and population health management. Specifically, this study used an agentic AI evidence-generation platform to obtain epidemiological estimates of diverse medical conditions, with the results evaluated against existing AI frameworks.

**Methods:**

Prevalence estimates of 6 conditions (amyotrophic lateral sclerosis, acute myeloid leukemia, bladder cancer, Huntington disease, elevated lipoprotein (a), and Parkinson disease) were estimated using an agentic AI evidence-generation platform applied to an administrative claims database with representation from every US state. Gender-specific rates were calculated within the following age categories: 0 to 17, 18 to 24, 25 to 34, 35 to 44, 45 to 54, 55 to 64, and 65 to 74 years and 75 years and older. Period prevalence was estimated from January 1, 2020, to June 30, 2025, and annual prevalence rates for each year were estimated from 2020 to 2024. Continuous enrollment for 12 months was required during the study period for inclusion. Source code generated by the platform as part of the analysis was reviewed by an independent programmer for validation of methods and programming, and analyses were replicated using traditional programming methods. Results obtained throughout the process were evaluated against several existing AI application frameworks.

**Results:**

Regarding accuracy, epidemiological estimates obtained using the agentic AI platform were consistent with published estimates for all 6 conditions, as well as with estimates obtained from traditional programming methods. Regarding rigor, the agentic AI platform conducted the analysis with rigor by confirming acceptable methods in published literature for the type of data source used. Code lists used for the analysis were confirmed against existing algorithms when available. Appropriate statistical methods were used to compare differences in prevalence rates by age and gender. Regarding trust (explainability, transparency, replicability, traceability, and validation), the agentic AI platform generated all source code used for the analyses, which was reviewed and validated for accuracy and appropriateness. The analysis included a “human in the loop” to validate the research question, data extraction method, statistical analysis plan, and output plan prior to proceeding with each step.

**Conclusions:**

With specific design considerations to ensure responsible use, agentic AI can be invaluable to increasing the accessibility of large datasets for applied clinical outcome research and population health management analyses.

## Introduction

There is tremendous enthusiasm for the use of AI in health care because of the ability to analyze existing data for preventative, diagnostic, and treatment support [1-4]. AI is a broad term and encompasses a diverse array of tools and functions. As AI continues to evolve, it is important to differentiate between AI agents, or generative AI, and agentic AI to understand the value and limitations of various applications in health care. AI agents have the ability to cull information from massive datasets using large language models and can generate content and answer questions, whereas agentic AI refers to systems capable of autonomous decision-making and self-learning to accomplish complex scientific tasks [5-8]. An example of differentiation is a literature review: generative AI can identify appropriate literature, whereas agentic AI can identify literature and apply relevant findings to adapt study methodology to conform to accepted standards.

Over the past decade, the recognition of the value of real-world evidence (RWE), defined as evidence generated during real-world patient care, has exploded [9,10]. RWE is generated from real-world data (RWD) sources, meaning data that are generated through the process of patient care, such as electronic health records, administrative claims and billing data, laboratory data, registries, patient monitoring devices, etc. The use of RWD to generate RWE has typically been limited to researchers with sophisticated programming skills capable of analyzing such large datasets, especially as the data were collected for other purposes, thereby requiring a significant amount of preparation and data cleaning for analytic purposes. For example, administrative claims and billing data represent a rich source of information on health care services provided as a claim is generated for practically every health care interaction. However, understanding how to use the data requires knowledge of medical coding, application of continuous enrollment criteria, and understanding of the claims adjudication process. This complexity can make the use of RWD difficult for health care and clinical applications such as clinical outcome monitoring and population health management.

AI, specifically agentic AI, can make access to large real-world datasets for the generation of RWE for health care and clinical applications feasible for health care providers, researchers, and administrators without access to large analytic programming resources. A recent survey of physician attitudes toward the use of AI in medicine found that acceptance and enthusiasm were shaped more by experience with and engagement in the use of AI than by demographic or physician characteristics [11]. AI is only as good as the data used to create the results [12]. Accountability and trust are also important factors [2,13,14]. Trust is multifactorial and encompasses fairness, transparency, robustness, and reliability. Moreover, while agentic AI has the capability to operate autonomously, having a “human in the loop” to confirm decisions at certain critical times during the process is important to ensuring accountability and trust [2,15].

The objective of this study was to understand the feasibility of using agentic AI for clinical outcome research and population health management evaluated against applicable frameworks of the responsible use of AI in health care. Specifically, this study used HealthVerity eXOs, an agentic AI evidence-generation platform powered by Medeloop, to obtain epidemiological estimates of several diverse medical conditions as subgroup identification is the foundation of clinical outcome research and population health management.

## Methods

### Study Design

Prevalence estimates of 6 diverse conditions were obtained using an agentic AI evidence-generation platform applied to an administrative claims database with representation from every US state. Using claims databases to estimate prevalence has been well documented in many disease areas [16-19]. Prevalence estimates were obtained for amyotrophic lateral sclerosis (ALS), acute myeloid leukemia (AML), bladder cancer, Huntington disease (HD), elevated lipoprotein (a), and Parkinson disease (PD). The *International Classification of Diseases, 10th Revision* (*ICD-10*), diagnosis codes used to define each of the conditions are available in Multimedia Appendix 1. The conditions were selected to represent diverse conditions across varying specialty areas and to include both common and rare conditions. Gender-specific rates were calculated within the following age categories: 0 to 17, 18 to 24, 25 to 34, 35 to 44, 45 to 54, 55 to 64, and 65 to 74 years and 75 years and older. Period prevalence was estimated from January 1, 2020, to June 30, 2025. A minimum of 12 months of continuous enrollment was required for inclusion as is typical in analyses using closed claims data. Clopper-Pearson, Wald, or Wilson 95% CIs, as appropriate, were computed for each stratum-specific prevalence estimate, and Pearson chi-square tests were conducted to test for differences within the strata. Statistical significance was assessed at an α value of .05.

### AI Validation

The following standardized prompt was used across analyses for consistency: “What is the period prevalence of <condition> (expressed as a rate per 100,000), and how do these rate differ according to these age categories (0–17, 18–24, 25–34, 35–44, 45–54, 55–64, 65–74, 75 and older) and gender within these age categories? Require continuous enrollment for a minimum of 12 months at any time during the study period.” The agentic AI platform allows for human-in-the-loop acceptance at the following steps of the analysis: query validation, data extraction plan, statistical plan, and output plan. The platform also conducts targeted literature reviews to identify accepted methodologies, including disease identification algorithms. All source code used for the analysis is also generated with the results. Source code was reviewed by an independent programmer for validation of methods and programming. The independent programmer was blinded to the creator of the source code and was not aware that it was generated by agentic AI. The programmer had direct experience with the dataset used for the analysis and was provided with the study objective of estimating the period prevalence of 6 diverse conditions, including the age and gender breakdown. The programmer was asked to review the code to determine whether the source code could meet the study objectives and used appropriate methods and to replicate the analysis using traditional programming methods.

The underlying architecture comprises a researcher agent (orchestrator), a semantic engine, an AI-powered notebook, and resources. The researcher agent interacts with the user to both receive and respond to questions. The semantic engine comprises an agent that has access to a semantic search querier and semantic database for concept extraction, code extraction, code lookup, and code retrieval. The researcher agent sends tasks to the AI-powered notebook, which comprises an agent that writes code and uses the notebook, which is connected to the cloud-based dataset, to complete tasks. The resources include custom logic and other reference files. The results from the semantic engine and AI-powered notebook are sent back to the researcher agent to coordinate all research tasks per the user-approved plan. The researcher agent conducts the final synthesis of information to present it back to the user. At the time of this study, fine-tuned versions of GPT-4.1 (OpenAI) and text-embedding-3-large were used for search and embeddings, respectively, to power the semantic engine.

### Ethical Considerations

Because this study used anonymized, deidentified data collected in the ordinary course of care, institutional review board approval and Declaration of Helsinki considerations were not required in accordance with Title 45 of the Code of Federal Regulations part 46.104(d)(4)(ii). All study data were handled through protocols compliant with HIPAA (Health Insurance Portability and Accountability Act).

## Results

The administrative claims data included 59 million individuals from January 1, 2020, to June 30, 2025, with every state in the United States represented. The demographics of the study population, as well as geographic region, are shown in Table 1.

Of the 59 million individuals in the study population, 38.4 million (65.1%) met the continuous enrollment requirement of 12 months during the study period. The demographics of the study population are similar to those of the US population. Although not used in the analysis, every geographic region of the United States was represented in the data, and the distribution of those with known race was similar to that of the population of the United States.

The overall period prevalence for each of the 6 conditions is shown in Table 2, and the age-gender breakdowns for each condition are illustrated in Figure 1.

As expected, period prevalence was higher than the annual prevalence estimates. There was a greater difference between annual prevalence and period prevalence estimates for conditions where there was likely to be less overlap in the patients represented in each year. Interestingly, there were differences in how the annual prevalences were calculated across conditions within the study period. For ALS, HD, elevated lipoprotein (a), and PD, an individual who was confirmed in the numerator in one year was carried forward to the numerators of subsequent years as all are lifelong diseases; however, annual prevalences were calculated independently without carrying forward for the other conditions. The differences in prevalence rates when calculated using traditional programming methods ranged from −1.0 to +0.1 in all instances and are listed in Multimedia Appendix 2.

**Table 1:** Retrospective US claims data study population characteristics for individuals in the dataset from January 1, 2020, to June 30, 2025 (N=38.4 million)^a^.

Characteristic		Patients (millions), n (%)
**Gender**		
	Female	20 (52.2)
	Male	17.5 (45.5)
	Other or unknown	0.9 (2.2)
**Age (y)**		
	0-17	8.9 (23.3)
	18-24	3.9 (10.2)
	25-34	5.2 (13.5)
	35-44	4.9 (12.8)
	45-54	4.3 (11.2)
	55-64	4.6 (12.0)
	65-74	3.4 (8.9)
	≥75	2.3 (6.0)
	Missing or unknown	0.9 (2.3)
**Race and ethnicity**		
	Asian	0.7 (1.8)
	Black	2.7 (7.0)
	White	11.1 (28.9)
	Other	0.9 (2.3)
	Missing or unknown	23.0 (59.9)
**Region**		
	Midwest	10.4 (27.1)
	Northeast	6.8 (17.7)
	South	11.8 (30.7)
	West	8.3 (21.6)
	Missing or unknown	1.1 (2.9)

^a^Percentages do not add up to 100 due to rounding.

**Table 2:** Overall period prevalence of 6 diverse conditions in a retrospective administrative US claims data analysis from January 1, 2020 to June 30, 2025.

Condition	Prevalence rate per 100,000 (95% CI)					
	Overall	2020	2021	2022	2023	2024
ALS^a^	16.1 (15.6-16.6)	7.1 (6.7-7.5)	9.0 (8.6-9.4)	10.0 (9.5-10.4)	10.5 (10.1-10.9)	11.0 (10.5-11.4)
AML^b^	51.6 (50.7-52.4)	41.1 (36.6-46.1)	49.9 (47.1-52.9)	46.1 (43.6-48.7)	31.8 (30.3-33.4)	28.9 (27.6-30.4)
Bladder cancer	228.0 (226.3-229.8)	144.7 (142.8-146.6)	132.1 (130.6-133.8)	136.0 (134.4-137.6)	131.9 (130.3-133.4)	132.3 (130.7-134.0)
HD^c^	10.2 (9.8-10.6)	6.7 (6.3-7.1)	7.4 (7.0-7.8)	7.9 (7.5-8.3)	8.4 (8.0-8.8)	9.4 (8.9-9.8)
Elevated lipoprotein (a)	173.9 (172.4-175.5)	47.7 (46.4-49.0)	76.0 (74.5-77.5)	100.9 (99.2-102.6)	130.6 (128.5-132.7)	167.0 (164.2-170.0)
PD^d^	333.8 (331.7-335.9)	206.1 (203.9-208.4)	232.0 (229.9-234.1)	263.0 (260.9-265.2)	273.8 (271.6-276.0)	288.9 (286.4-291.4)

^a^ALS: amyotrophic lateral sclerosis.

^b^AML: acute myeloid leukemia.

^c^HD: Huntington disease.

^d^PD: Parkinson disease.

**Figure 1:**
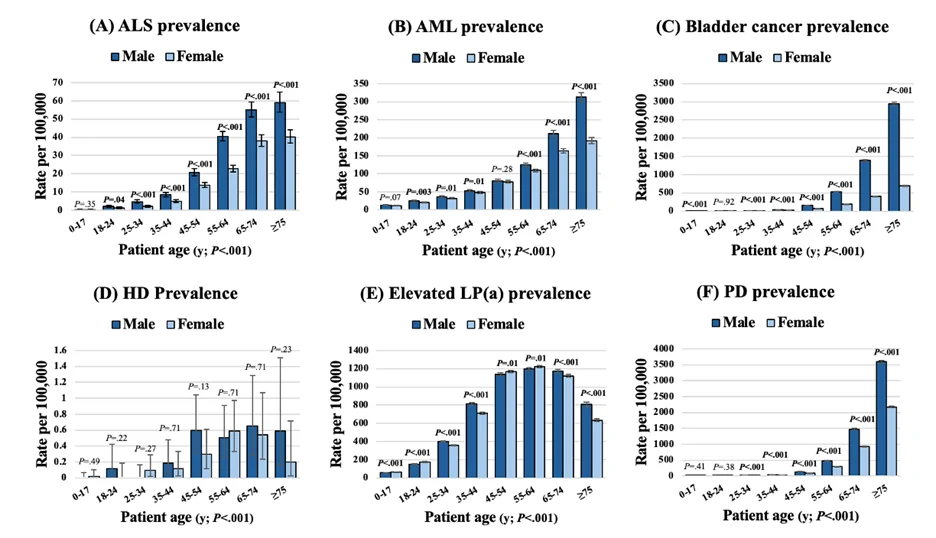
Period prevalence of 6 diverse conditions in a retrospective administrative US claims data analysis from January 1, 2020, to June 30, 2025, by gender and age category. *Statistically significant difference for age and gender comparisons; statistical significance assessed at α=.05. ALS: amyotrophic lateral sclerosis; AML: acute myeloid leukemia; HD: Huntington disease; LP(a): lipoprotein (a); PD: Parkinson disease.

For the gender- and age-specific estimates, there were statistically significant differences among the age groups for all conditions. The period prevalence of ALS, AML, bladder cancer, and PD was significantly higher in male than in female individuals in adults except for the ages of 18 to 24 years in bladder cancer and PD as well as the ages of 45 to 54 years in AML. The rarity of HD made gender differences within age groups difficult to detect. The prevalence of elevated lipoprotein (a) was similar and highest among the age groups of 45 to 74 years and then decreased at the ages of 75 years and older. The largest difference in gender was observed in the 35 to 44 years and 75 years and older age groups, where prevalence was significantly higher in male individuals. While statistically significant, female individuals had a slightly higher prevalence of elevated lipoprotein (a) than male individuals in the age groups of less than 25 years and 45 to 64 years.

With regard to human in the loop, there were 8 instances in which modifications to the specific analyses being suggested by the agentic AI were made to ensure consistency across the analyses. Table 3 shows the 8 instances of the analysis that were proposed by the agentic AI platform and the changes made during the human-in-the-loop confirmations of the proposed analyses.

**Table 3:** Human-in-the-loop changes to the analyses proposed by the agentic AI for the retrospective administrative US claims data prevalence analyses from January 1, 2020, to June 30, 2025.

Condition	Proposed by AI	Modification made	Stage in the process
Bladder cancer and Huntington disease	Continuous enrollment definition of a >45-d gap	Continuous enrollment definition of a >30-d gap	Acceptance of the statistical plan
Elevated lipoprotein (a) and acute myeloid leukemia	Calculation of prevalence rates for gender groups and age groups separately	Calculation of gender groups within each age subgroup	Validation of analysis question
Bladder cancer	C65.x, C66.x, and C67.x codes to align with the work by Gitlin et al [20]	Removal of C65.x and C66.x codes to align with clinical expert input	Acceptance of data extraction plan
Parkinson disease	G20, G20.Ax, G20.Bx, and G20.C codes	G20, G20.Ax, and G20.Bx codes	Acceptance of data extraction plan
Elevated lipoprotein (a) and Parkinson disease	No carryforward in estimating annual prevalence rates from 2020 to 2024	Specified carryforward to be used in estimating annual prevalence rates from 2020 to 2024	Acceptance of the statistical plan

Two instances were related to the definition of an acceptable gap allowed for the implementation of continuous enrollment criteria and were edited prior to acceptance of the statistical analysis plan, 2 instances were related to inconsistent interpretation of producing age-gender breakdowns of prevalence rates and were edited prior to the acceptance of the query validation, 2 instances involved the modification of the codes used to identify the target population and were edited prior to the acceptance of the data extraction plan, and 2 instances required the addition of the specification to use a carryforward approach to calculate the annual prevalence estimates (elevated lipoprotein (a) and PD). The selection of continuous enrollment criteria to be defined by less than a 45-day gap in enrollment as opposed to 30 days was not wrong per se but was modified for consistency across the 6 conditions. Both 30- and 45-day definitions are commonly used for acceptable gaps in enrollment to still be considered continuously enrolled. Although prompts for each condition used the exact same wording, there were 2 instances in which the subgroups were not interpreted correctly to be gender groups *within* the age groups. These instances were corrected with further clarification at the validation of the analysis question. The 2 instances in which the selection of the *ICD-10* coding was updated were based on clinical expert input. For bladder cancer, the code list initially selected was based on a published bladder cancer study using administrative claims data. There were several studies published that all had different code lists for the selection of the study population. The clinical expert advised that the lists were not wrong but would result in a narrower definition with a higher sensitivity for identifying those with the condition. For PD, the code changed in late 2023 from G20 with no modifiers to having 5 different modifiers. G20.C represented parkinsonisms. The clinical expert felt that a patient could have parkinsonisms without having a diagnosis of PD, thus advising to exclude that code. The agentic AI platform opted to run a sensitivity analysis with and without the code given the uncertainty of the inclusion in defining PD. Finally, when initially running the inquiry for elevated lipoprotein (a) and PD, annual estimates were not calculated using a carryforward approach. Because PD is a chronic condition with no current “cure” and elevated lipoprotein (a) is a genetic marker, use of the carryforward approach was appropriate for these conditions.

Due to the size of some of the analyses based on the number in the target population, some analyses were broken down and conducted in several iterations on the platform. In these instances, the overall period prevalence estimates were calculated separately from the gender and age category breakdown.

## Discussion

### Principal Findings

Epidemiological estimates across 6 diverse conditions were calculated using both traditional programming methods and an agentic AI platform that included a human in the loop to validate the analysis at several key times throughout. The estimates that were generated were practically the same and were completed in much less time (ie, approximately 80%-90% less time on average) using the agentic AI platform.

### Application of Relevant Frameworks Addressing the Use of AI in Health Care

Several frameworks have emerged to provide guidance for the responsible use of AI in health care [2,4,12,21]. These frameworks can be used to understand the feasibility of using agentic AI for clinical outcome research and population health management.

#### Accuracy

Prevalence estimates can vary widely for a variety of reasons: representativeness and accuracy of the data source used to calculate, methodology used to estimate, period, geography, etc [22-24]. This study produced prevalence estimates that were broadly consistent with previously published estimates for all the conditions, including gender and age differences [25-40]. Given the difficulty of replicating published prevalence estimates in differing data sources, this study also estimated prevalence through traditional programming methods using the same administrative claims dataset. The results were consistent with the estimates obtained using the agentic AI platform.

While there were 8 instances in which human intervention was necessary to achieve the desired analysis, this study was unique in that consistency across individual analyses was important. The agentic AI platform for continuous enrollment criteria and diagnosis code selection was based on published literature. The choices were scientifically acceptable options, although they were not what was preferred for *this* study. Two of the instances dealt with correcting the interpretation of the prompt. The desired analysis was to calculate prevalence of gender within age subgroups but was interpreted as gender and age subgroups despite having the exact same prompt wording across conditions. Additionally, for the 4 conditions in which carryforward methodology was appropriate when calculating serial annual prevalences, 2 instances had to be corrected to specify using carryforward. That is why having a human in the loop is necessary to validate the analysis. In many instances, there are several options that are equally valid, such as continuous enrollment criteria; in other instances, nuances of methodology specific to the subject area need to be confirmed. The user may have specific reasons for choosing one method over another. Thus, the human in the loop ensures that the analysis approach meets the specific needs and preferences of the user.

#### Rigor

Identifying individuals with specific conditions and estimating disease prevalence are the foundation for quantifying burden, managing population health, and evaluating clinical outcomes [4]. While some may consider this type of analysis descriptive, there is a need for rigor as there are factors that must be accounted for in the analysis that make the use of agentic AI valuable. Depending on the data source, there are accepted methods for estimating prevalence [23,24,41]. For example, when using closed claims data, continuous enrollment requirements are standard practice as this ensures that an individual is represented in the data for sufficient time to be able to be identified for the numerator of the prevalence rate. Prevalence rates calculated using closed claims data that do not use continuous enrollment criteria will most likely underestimate the true prevalence. Before conducting the analysis, the agentic AI platform used in this study performed a targeted literature review to verify that the analytical methods selected were established and appropriate for addressing the specific research question.

Another example of potential bias in estimating prevalence using administrative claims data stems from the use of coding to identify the condition. Identifying some conditions is straightforward when there are clear diagnosis codes that are used. Prior to late 2023, PD was a condition with G20 *ICD-10* diagnosis codes representing all its various presentations. Diagnosis codes are created to represent conditions and relevant variations of them for reimbursement. An example where coding is not as straightforward to capture a condition is bladder cancer. *ICD-10* C67.* codes represent malignant neoplasm of the bladder. However, a recent study defined bladder cancer to also include C65.* (malignant neoplasm of the renal pelvis) and C66.* (malignant neoplasm of the ureter) codes with C67.* codes [20]. Other relevant codes include C79.1* (secondary malignant neoplasm of the bladder and other and unspecified urinary organs), D41.4 (neoplasm of uncertain behavior of the bladder), and D09.0 (carcinoma in situ of the bladder). Differences in the inclusion or exclusion of codes will influence the prevalence estimate obtained using administrative claims data. The agentic AI platform used for this study generates a code list that will be used, conducts a targeted literature search to identify algorithms for identification that have been used in previous studies, and allows the user to edit the list prior to implementation in the analysis.

#### Trust

Trust is a broad concept and is often represented by factors such as explainability, transparency, reliability, traceability, and validation. These factors can be challenging with agentic AI given the autonomous nature of the technology. Having a human in the loop for collaboration is essential to building trust. The specific agentic AI platform used for the analysis pauses at various points in the process to give the user the ability to edit. Key steps in which having a human in the loop are critical are (1) the validation of the overall analysis prompt (ie, was the prompt interpreted as intended?), (2) the data extraction process and creation of the analytic data file (ie, what criteria will be used to create the analytic data file?), (3) generation of the statistical analysis plan, and (4) creation of the output plan (ie, how will results be presented?). Providing input into these critical steps in the process can ensure that the results are clinically relevant, explainable, and scientifically appropriate.

Even with the human in the loop at critical steps in the process, there are a myriad of decisions that are made during processing, including the order in which steps are taken, that can influence the specific results obtained. Being able to view the specific source code that is used throughout the process is the only way to have the ability to replicate the analysis fully. This level of transparency is essential for validation and reliability. For this study, an independent programmer reviewed all source code generated from the agentic AI platform to validate the methods and process used and was able to produce the results through traditional programming methods.

There are some limitations to using agentic AI to conduct RWD analyses. Understanding how to develop appropriate prompts that result in the desired analysis takes some iteration if the user is not experienced in using agentic AI. Most people think of generative AI when they hear “AI.” Previous research has shown that comfort with using AI increases with experience [11]. Using agentic AI to conduct analyses do not provide instantaneous results.

### Comparison to Traditional Programming Methods

While not as fast as generative AI, agentic AI in RWD analyses is significantly faster than traditional programming methods. An analysis from the start of data extraction, creation of the analytic file, and conduct of the analysis would take an experienced programmer a minimum of 20 programming hours for each condition and includes targeted literature reviews, code list validation, and protocol generation. The agentic AI platform was able to complete analyses of conditions in between 40 and 90 minutes depending on the complexity of the identification of the condition and the size of the patient population with that condition.

When considering the time and resources required to conduct a comprehensive epidemiological estimate that includes targeted literature review, validation of coding, creation of the analytic file, and programming, it is easy to understand the potential in efficiency that using an agentic AI platform for such analyses can bring.

Regardless of increases in efficiency, stakeholders who are interested in conducting such analyses may not have the expertise or resources necessary to conduct a comprehensive analysis. Consequently, analyses of large datasets may not be feasible for clinical outcome research and population health management. The ability to use an agentic AI platform makes such analyses feasible for many stakeholders that would benefit from generating evidence that can be used for clinical decision-making and population health management.

### Limitations

This study has several limitations. First, in understanding the application of agentic AI for clinical outcome research and population health management, generating epidemiological estimates was selected to represent these broad types of research and real-world use as most begin by identifying and estimating the population of interest. However, there are many types of research studies and analyses for which this technology is applicable. In addition to epidemiological estimates, other analyses in which it may be valuable to use an agentic AI platform include clinical practice population health management, use and treatment patterns, clinical comparative effectiveness, quality improvement, assessment of guideline-directed care, adherence and persistency with treatment, clinical trial design and feasibility, and understanding the patient health care journey. Second, it is difficult to evaluate the accuracy of the results as so many factors can influence epidemiological estimates, such as data source, time frame, continuous enrollment criteria, criteria for numerator identification. Results compared with published estimates had broad face validity; however, statistical comparison was not made as differences may be based on other factors and not on the actual accuracy of the agentic AI. Third, this study was limited to analyses using a large administrative claims database. The value in the use of agentic AI for other types of data, such as electronic health records, registries, clinical trials, etc, may not be generalizable to these datasets and should be explored in future research. Another limitation that is common to most administrative datasets is the lack of completeness of race and ethnicity data. While the distribution of race and ethnicity in our study population seemed to be similar to that of the general population, over half of the patients in the dataset were missing race and ethnicity. The study design of only requiring a period of 12 months of continuous enrollment at any time during the study period of over 5 years was intended to maximize the number of patients that could be identified. On the other hand, this study design could have underestimated prevalence if the denominator was inflated. Finally, the agentic AI platform was specifically designed for health outcome research. Other agentic AI platforms may not produce similar results in these types of studies and analyses.

### Conclusions

With specific design considerations to ensure responsible use, agentic AI can be invaluable to making large datasets accessible for applied clinical outcome research and population health management analyses.

## Data Availability

The dataset analyzed for this study is not publicly available and is accessible via subscription only. Data and source code for the analyses for verification purposes can be made available from the corresponding author on reasonable request.

## Appendix

Multimedia Appendix 1 International Classification of Diseases, 10th Revision, diagnosis codes used to identify patients in the retrospective administrative US claims database analysis of prevalence from January 1, 2020, to June 30, 2025.

Multimedia Appendix 2 Comparison of overall prevalence in administrative US claims data from January 1, 2020, to June 30, 2025, using traditional programming methods and an agentic AI platform.

## Abbreviations

ALS: amyotrophic lateral sclerosis
AML: acute myeloid leukemia
HD: Huntington disease
HIPAA: Health Insurance Portability and Accountability Act
ICD-10: International Classification of Diseases, 10th Revision
PD: Parkinson disease
RWD: real-world data
RWE: real-world evidence
