# Supplementary material for: Agentic AI for Clinical Outcomes Research, Population Health Management Analyses With Large Administrative Databases, and Generating Epidemiological Estimates of Diseases: Feasibility and Validation Study
Source: J Med Internet Res. 2026 Sep 30;28:e100284. doi: 10.2196/100284 (PMC13669707; doi:10.2196/100284)
Supplement: Supplementary file 1 [file jmir_v28i1e100284_app1.docx]

**Appendix A. ICD-10 diagnosis codes used to identify patients in the retrospective administrative U.S. claims database analysis of prevalence from Jan 1, 2020 – Jun 30, 2025**

| Condition | ICD-10 diagnosis codes |
| --- | --- |
| Amyotrophic lateral sclerosis (ALS) | G12.21 |
| Acute myeloid leukemia (AML) | C92.0x, C92.4x, C92.Ax, C92.6x, C92.5x, C93.0x, C93.91, C94.2x, C94.4x |
| Bladder cancer | C67.x |
| Huntington’s disease | G10 |
| Elevated lipoprotein (a) (LP(a)) | E78.41 |
| Parkinson’s disease | G20, G20.Ax, G20.Bx |
