# Supplementary material for: Agentic AI for Clinical Outcomes Research, Population Health Management Analyses With Large Administrative Databases, and Generating Epidemiological Estimates of Diseases: Feasibility and Validation Study
Source: J Med Internet Res. 2026 Sep 30;28:e100284. doi: 10.2196/100284 (PMC13669707; doi:10.2196/100284)
Supplement: Supplementary file 2 [file jmir_v28i1e100284_app2.docx]

**Appendix B. Comparison of overall prevalence in administrative U.S. claims data from Jan 1, 2020 – Jun 30, 2025 using traditional programming methods and an agentic AI platform**

| Condition | Overall prevalence using traditional programming method | Overall prevalence as calculated in the agentic AI platform | Absolute difference |
| --- | --- | --- | --- |
| ALS | 16.6  (16.1-17.01) | 16.1  (15.6-16.6) | -0.5 |
| AML | 52.4  (51.6-53.3) | 51.6  (50.7-52.4) | -0.8 |
| Bladder cancer | 229.0  (227.2-230.8) | 228.0  (226.3–229.8) | -1.0 |
| HD | 10.4  (10.1-10.8) | 10.2  (9.8-10.6) | -0.2 |
| Elevated LP(a) | 174.1  (172.6-175.7) | 173.9  (172.4–175.5) | -0.2 |
| PD | 333.7  (331.6-335.8) | 333.8  (331.7-335.9) | +0.1 |

ALS=amyotrophic lateral sclerosis; AML=acute myeloid leukemia; HD=Huntington’s disease; LP(a)=lipoprotein(a); PD=Parkinson’s disease
